# SMA Identified: Clinical and Molecular Findings From a Sponsored Testing Program for Spinal Muscular Atrophy in More Than 2,000 Individuals

**DOI:** 10.3389/fneur.2021.663911

**Published:** 2021-05-06

**Authors:** B. Monica Bowen, Rebecca Truty, Swaroop Aradhya, Sara L. Bristow, Britt A. Johnson, Ana Morales, Christopher A. Tan, M. Jody Westbrook, Thomas L. Winder, Juan C. Chavez

**Affiliations:** ^1^Invitae, San Francisco, CA, United States; ^2^Biogen, Cambridge, MA, United States

**Keywords:** spinal muscular atrophy, neuromuscular disorders, genetics, inherited neurologic disorders, motor neuron disease

## Abstract

**Background:** Spinal muscular atrophy (SMA) linked to chromosome 5q is an inherited progressive neuromuscular disorder with a narrow therapeutic window for optimal treatment. Although genetic testing provides a definitive molecular diagnosis that can facilitate access to effective treatments, limited awareness and other barriers may prohibit widespread testing. In this study, the clinical and molecular findings of SMA Identified—a no-charge sponsored next-generation sequencing (NGS)-based genetic testing program for SMA diagnosis—are reported.

**Methods:** Between March 2018 and March 2020, unrelated individuals who had a confirmed or suspected SMA diagnosis or had a family history of SMA were eligible. All individuals underwent diagnostic genetic testing for SMA at clinician discretion. In total, 2,459 individuals were tested and included in this analysis. An NGS-based approach interrogated sequence and copy number of *SMN1* and *SMN2*. Variants were confirmed by multiplex ligation-dependent probe amplification sequencing. Individuals were categorized according to genetic test results: diagnostic (two pathogenic *SMN1* variants), nearly diagnostic (*SMN1* exon-7 deletion with a variant of uncertain significance [VUS] in *SMN1* or *SMN2*), indeterminate VUS (one VUS in *SMN1* or *SMN2*), carrier (heterozygous *SMN1* deletion only), or negative (no pathogenic variants or VUS in *SMN1* or *SMN2*). Diagnostic yield was calculated. Genetic test results were analyzed based on clinician-reported clinical features and genetic modifiers (*SMN2* copy number and *SMN2* c.859G>C).

**Results:** In total, 2,459 unrelated individuals (mean age 24.3 ± 23.0 years) underwent diagnostic testing. The diagnostic yield for diagnostic plus nearly diagnostic results was 31.3% (*n* = 771/2,459). Age of onset and clinical presentation varied considerably for individuals and was dependent on *SMN2* copy number. Homozygous deletions represented the most common genetic etiology (96.2%), with sequence variants also observed in probands with clinical diagnoses of SMA.

**Conclusions:** Using a high-yield panel test in a no-charge sponsored program early in the diagnostic odyssey may open the door for medical interventions in a substantial number of individuals with SMA. These findings have potential implications for clinical management of probands and their families.

## Introduction

Spinal muscular atrophy (SMA) is a progressive neuromuscular disorder that is the most common inherited cause of death in infants. Its estimated incidence ranges from 5 to 13 per 100,000 individuals, varying based largely on ancestry (1). SMA is characterized by the loss of alpha motor neurons in the spinal cord, resulting in progressive muscle atrophy, weakness, and ultimately paralysis. Other features of SMA include muscle fasciculations, tremor, poor weight gain, sleeping difficulties, pneumonia, scoliosis, joint contractures, and congenital heart disease. Depending on the severity of symptoms and age of symptom onset, SMA diagnosis is classified into one of four groups, ranging from severe with infantile onset leading to death in childhood (Type I) to mild with delayed onset of symptoms until puberty or later (Type IV). Although those with severe forms of SMA are diagnosed early, many affected individuals with milder forms of the disorder are not diagnosed until adulthood (2, 3).

The most common form of SMA is caused by biallelic pathogenic variants in *SMN1*. The vast majority of SMA molecular diagnoses (~95%) are due to homozygous deletion of the entire *SMN1* gene, while a minority (~5%) are due to a compound heterozygous sequence variant in *SMN1* on one chromosome and an *SMN1* gene deletion on the other chromosome (4). Disease severity and progression are modulated by *SMN2*, a paralog of *SMN1* differing from *SMN1* by 10 base pairs. One of the sequence differences results in 90% reduced pre-mRNA processing of *SMN2*. Thus, increased copy number of *SMN2* can modulate the loss of SMN1 protein (5, 6). Another genetic modifier in *SMN2* is the c.859G>C variant located in exon 7. Several studies have demonstrated that affected individuals with a cytosine at this position have milder symptoms, even when only two copies of *SMN2* are present (7, 8).

There are several challenges in providing a molecular diagnosis to individuals with SMA. The coding regions of *SMN1* and *SMN2* differ by a single nucleotide variant at position c.840 in exon 7, commonly referred to as the gene-determining variant, which is used to determine *SMN1* and *SMN2* copy number. A next-generation sequencing (NGS)-based method has been developed that enables simultaneous sequencing and copy number analysis of *SMN1* and *SMN2* and addresses inherent limitations of traditional SMA testing methods (9–12). However, long-range (LR)-PCR is still required to distinguish single nucleotide variants in *SMN1* from those in *SMN2*.

Several therapies have become available for SMA in recent years (13–15). However, there is a narrow therapeutic window for optimally impacting progression of SMA in the incident population; therefore, early molecular diagnoses are critical in managing patient care (16). In May 2018, Biogen and Invitae introduced the SMA Identified sponsored testing program to provide molecular diagnostic testing at no charge for individuals with clinical or suspected diagnoses of SMA (17). Here, we investigate the diagnostic yield from SMA Identified. To better understand the clinical presentation of SMA, we evaluate correlations between clinical features and positive molecular diagnoses.

## Methods

### Study Population

Individuals who received testing between March 2018 and March 2020 through the SMA Identified sponsored testing program were eligible for study inclusion. SMA Identified is open to all individuals with a diagnosis, suspected diagnosis, or family history of SMA receiving testing in the United States. Only unrelated individuals who underwent diagnostic testing for SMA were included in this study. Family members of these unrelated individuals who underwent diagnostic testing (*n* = 151) and asymptomatic individuals with a family history who underwent carrier screening (*n* = 39) who were tested through the program were excluded from this analysis. Ordering clinicians reported the presence of the following clinical features on an intake form for all individuals undergoing testing: muscle weakness (symmetrical, proximal greater than distal, and/or greater in the legs than in the arms), respiratory issues, bulbar dysfunction, scoliosis, joint contractures, tongue fasciculations, absent or diminished tendon reflexes, spinal rods, and spinal fusions.

All patients provided informed consent for genetic testing as well as for sharing their de-identified data for research purposes. This study was approved by the Western Institutional Review Board.

### Gene Panels

Among individuals receiving testing through SMA Identified who were included in this study, the ordering clinician initially selected one of two available SMA tests depending on the individual's needs: the Spinal Muscular Atrophy Panel or the Spinal Muscular Atrophy STAT Panel. Both tests were used to analyze *SMN1* copy number and to analyze *SMN2* copy number in cases of *SMN1* homozygous or compound heterozygous deletions. The Spinal Muscular Atrophy Panel (but not the STAT panel) was also used to analyze sequence variants in *SMN1*. Individuals who were tested through the Spinal Muscular Atrophy Panel and received a negative result could subsequently order testing for the Comprehensive Neuromuscular Disorders Panel.

### Genetic Testing

All individual samples were extracted, sequenced, and analyzed in Invitae's accredited and certified molecular diagnostic laboratory as described previously (11, 12). The NGS-based Spinal Muscular Atrophy Panel sequenced *SMN1* and *SMN2* at high-depth coverage (50 × minimum, 350 × average). When the Spinal Muscular Atrophy STAT Panel was ordered, multiplex ligation-dependent probe amplification sequencing (MLPA-seq) was run to detect copy number.

*SMN1* and *SMN2* analyses were performed using a validated bioinformatic approach that accounted for the high sequence homology between *SMN1* and *SMN2* (11). Briefly, combined reads from both *SMN1* and *SMN2* were aligned to the *SMN1* reference sequence (NM_000344.3), and combined *SMN1/SMN2* copy number was determined using CNVitae, an NGS-based copy number variant detection algorithm (18, 19). *SMN1*- and *SMN2*-specific exon 7 copy number was resolved by analyzing the ratio of C to T at the c.840C>T gene-determining variant in exon 7. Sequence variants could be unambiguously assigned to either *SMN1* or *SMN2* only for exon 7 using the gene-determining variant.

All sequence and copy number variants were interpreted using the proprietary Sherloc statistical framework (20) to assign a variant classification of pathogenic (P), likely pathogenic (LP), variant(s) of uncertain significance (VUS), likely benign, or benign as specified by the joint consensus from the American College of Medical Genetics and Genomics and the Association for Molecular Pathology (21). Variants that were ambiguously in *SMN1* or *SMN2* were defined as VUS due to the high sequence homology. Specifically for *SMN1* and *SMN2*, P/LP copy number variants were confirmed by an orthogonal method (MLPA-seq) (22, 23). Variants categorized as P/LP and VUS were reported to clinicians.

### Data Analysis

Individuals were categorized into one of five groups according to their genetic test results: diagnostic (two P/LP *SMN1* variants), nearly diagnostic (*SMN1* deletion with a VUS), indeterminate VUS (one VUS), carrier (heterozygous *SMN1* deletion only), or negative (no P/LP variants or VUS in *SMN1* or *SMN2*). A positive molecular diagnosis was defined as a diagnostic or nearly diagnostic result.

Diagnostic yield was calculated. Additionally, the association of each clinical feature with SMA genetic testing was assessed by calculating the proportions of individuals in the diagnostic, nearly diagnostic, indeterminate VUS, and carrier groups who reported each clinical feature.

Among individuals with a positive molecular diagnosis, *SMN2* copy number was determined and correlated with age at the time of testing and number of clinician-reported symptoms. In addition to investigating *SMN2* copy number, the putative modifier variant *SMN2* c.859G>C was assessed for its relationship to *SMN2* copy number and age at time of testing among individuals with a positive molecular diagnosis. Because of the low prevalence of this variant [gnomAD frequency 0.3% (24)], we evaluated it in a pooled cohort of individuals tested at Invitae either through or outside of the SMA Identified sponsored program. This variant is not routinely reported in clinical reports but was analyzed in this cohort to understand its potential role in modifying disease severity.

The frequencies of reported clinical features were assessed and compared between individuals with diagnostic or nearly diagnostic results with sequence variants and those with diagnostic results of homozygous *SMN1* deletion. In addition, pedigrees were analyzed in cases where sufficient clinical information was provided to understand nuances in symptoms and variant phasing.

All statistical analyses were performed using R version 3.4.2 (25). Where appropriate, the 95% confidence interval was calculated using the prop.test function in R. Differences in age using Welch's two-sample *t*-test between those homozygous for *SMN1* deletion or those with one *SMN1* deletion with a sequence variant. Spearman's rho was used to assess the correlation of copy number with patient age, and a two-way ANOVA was used to assess the impact of the modifier allele and *SMN2* copy number on patient age (alpha = 0.05). Pedigrees were drawn using Visual Paradigm (https://online.visual-paradigm.com/).

## Results

### Diagnostic Yield

**Figure 1:**
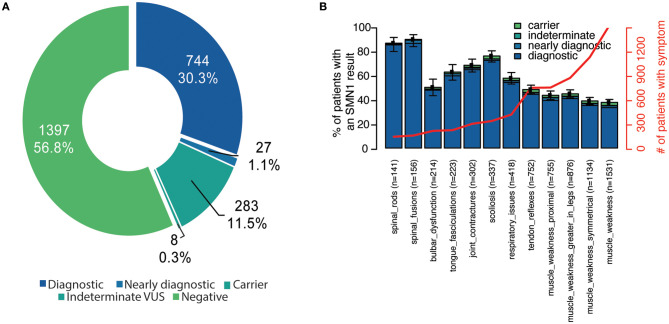
Diagnostic yield and reported clinical features among unrelated individuals in the SMA Identified program. **(A)** Individuals were stratified according to their genetic testing result. Diagnostic indicates that the individual had two P/LP *SMN1* variants in trans (homozygous deletion or compound heterozygous P/LP). Nearly diagnostic individuals had a heterozygous deletion of *SMN1* in combination with either a VUS unambiguously in *SMN1* or an ambiguous variant in *SMN1* or *SMN2*. Indeterminate VUS results were defined as one VUS. Carriers were heterozygous for the *SMN1* deletion. Those categorized as negative had no P/LP *SMN1* or *SMN2* variants detected (even if they had a P/LP variant in another gene if tested through a multi-gene panel). **(B)** For each clinician-reported clinical feature, the proportion of individuals with the symptom was also calculated (number of individuals indicated in x-axis and red line). Those with a negative result represent the remaining space above each stacked bar. Bar indicates 95% confidence interval for each clinical feature. P/LP, pathogenic/likely pathogenic; SMA, spinal muscular atrophy; VUS, variant of uncertain significance.

In total, 2,459 unrelated individuals (mean age 24.3 ± 23.0 years, range <1–89 years) underwent diagnostic testing through the SMA Identified sponsored program. The majority of individuals were aged 5 years or younger (Supplementary Figure 1). A positive result was observed in 771 individuals (744 diagnostic and 27 nearly diagnostic), resulting in a diagnostic yield of 31.3% (95% CI, 29.5–33.2%) (Figure 1A). These individuals ranged in age from 0 to 77 years (mean 25.7 ± 18.4 years).

Additional testing for genes other than *SMN1* and *SMN2* was ordered for 528 individuals in the cohort. Among those, five were diagnostic and one was nearly diagnostic for SMA. A positive or potentially positive result for another gene was returned 84 (15.9%) of individuals. The most common molecular diagnoses included findings in *DMD* (*n* = 8), *PMP22* (*n* = 7), *LMNA* (*n* = 7), and *RYR1* (*n* = 6).

### Clinical Presentations Observed in SMA Identified

Among all individuals who received testing through the sponsored program, the majority (64%) were reported to have two or more symptoms at the time of testing, and fewer than half (49%) were reported to have more than three. Muscle weakness, symmetrical muscle weakness, muscle weakness greater in the legs, and proximal muscle weakness were the most commonly reported symptoms in the cohort (Figure 1B).

Among individuals with a positive molecular diagnosis consistent with SMA, 74% had two or more and 65% had three or more clinician-reported symptoms. Spinal fusions, spinal rods, and scoliosis were most commonly associated with a positive molecular diagnosis, though, least commonly noted by clinicians (Figure 1B). Among those with a negative genetic test result, the most common reported individual clinical features included muscle weakness, reduced tendon reflexes, and bulbar dysfunction (Figure 1B).

When stratified by age, slight differences were observed in clinician-reported symptoms and their associations with a positive SMA diagnosis (Supplementary Figure 2). Among children aged <1 year, muscle weakness, symmetrical muscle weakness, and tendon reflexes were most commonly reported, while children reporting scoliosis, tongue fasciculations, and muscle weakness greater in the legs had the highest rate of positive molecular diagnosis. All of the clinical features were highly predictive of a positive SMA diagnosis in individuals aged 12–17 years and those aged 18–34 years. Spinal rods and spinal fusions were most common in SMA-positive individuals aged 35–64 years.

### Distribution of Sequence Variants

**Figure 2:**
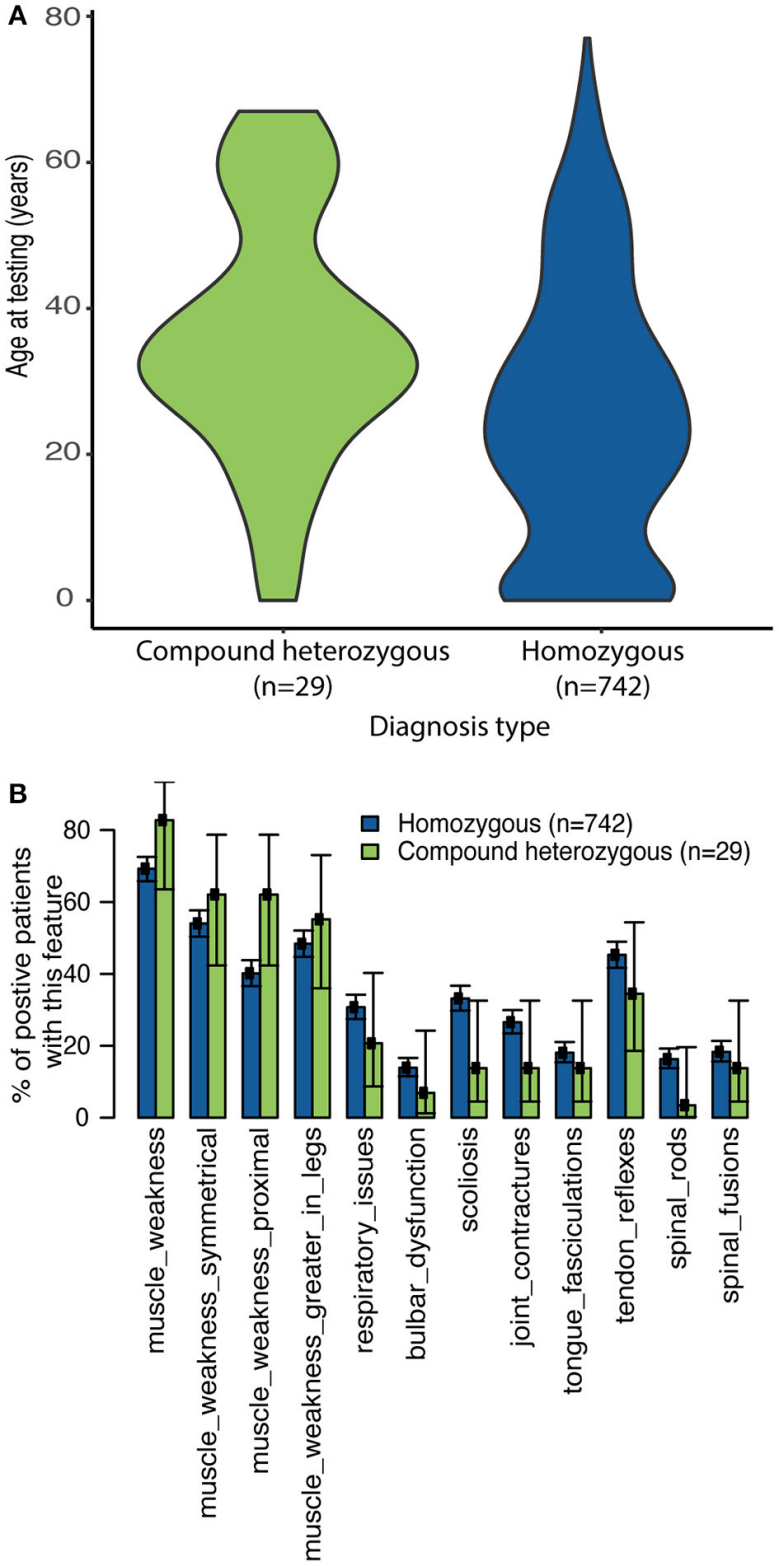
Clinician-reported clinical features among diagnostic and nearly diagnostic individuals tested through SMA Identified. **(A)** Violin plots reporting age at time of testing based on genotype. **(B)** The proportion of individuals with homozygous *SMN1* deletion or a single *SMN1* deletion plus either a P/LP variant or VUS on *SMN1* or *SMN2* who reported each clinical feature (as reported by the clinician) was calculated. 95% confidence intervals reported.

As expected, the classic homozygous deletion of *SMN1* exon 7 accounted for 96.2% (*n* = 742) of positive molecular diagnoses identified within the sponsored program, with 17 other variants accounting for the remaining 29 individuals with diagnostic (*n* = 2), or nearly diagnostic (*n* = 27) results (Supplementary Table 1). Individuals with sequence variants were significantly older at time of testing compared to those homozygous for the *SMN1* exon 7 deletion (35.7 vs. 25.4 years, *p* = 0.003232, Figure 2A). Phase was resolved for 11 (38%) compound heterozygotes, which included eight (28%) who had additional family testing supporting that the variant was likely in trans from the *SMN1* deletion and three (10%) who had NGS data confirming that the variant was located unambiguously in *SMN1*.

**Figure 3:**
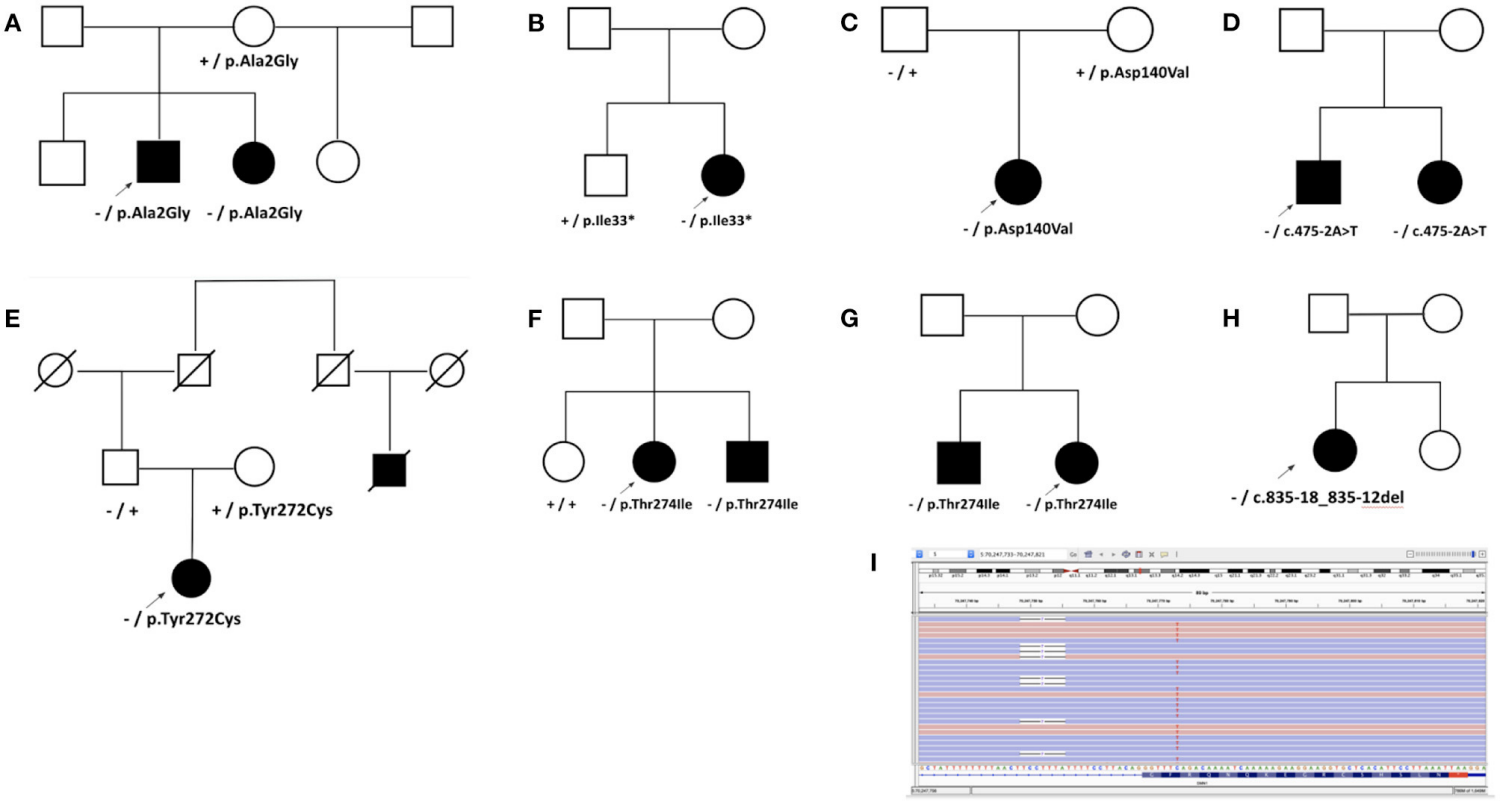
Analysis of pedigrees. Pedigrees were developed for families with nearly diagnostic sequence variants identified in probands undergoing SMA testing when additional clinical information was provided. Heterozygous deletion of *SMN1* is noted by “–,” while “+” denotes wild type. Sequence variants are noted by HGVS nomenclature. **(A)** Family A with p.Ala2Gly. The proband presented with delayed ambulation in childhood, had progressive weakness, and became wheelchair bound by his teenage years. His sister had similar muscle weakness as well as both variants. A brother and a maternal half-sister reported no weakness but were not available for testing. **(B)** Family B with p.Ile33*. The proband had proximal weakness onset in teenage years. In her unaffected brother, only p.Ile33* was detected. **(C)** Family C with p.Asp140Val. **(D)** Family D with c.475-2A>T. Disease onset occurred in the proband's teenage years. His sister also had progressive weakness and the same two variants. **(E)** Family E with p.Tyr272Cys. The proband presented with diffuse hypotonia, absent reflexes, and fasciculations. A paternal cousin, now deceased, was also reportedly affected. Both parents were unaffected. **(F)** Family F with p.Thr274Ile. The proband developed symptoms of difficulty rising from a chair in her early twenties, followed by slowly progressive lower and upper extremity weakness thereafter. She was diagnosed with SMA based on a muscle biopsy, and her brother was diagnosed as well after he developed similar symptoms. **(G)** Family G with p.Thr274Ile. **(H)** Family H with diagnostic 7-bp deletion c.835-18_835-12del in *SMN1* identified in the proband with SMA who also carried a heterozygous deletion of *SMN1*. **(I)** Sequencing reads in Integrative Genomics Viewer (IGV) showing c.835-18_835-12del opposite to the gene-determining variant in *SMN2*, c.840C>T, thus confirming the 7-bp deletion was in *SMN1* and in trans from the heterozygous deletion of *SMN1* in the Family H proband.

In general, the reported frequency of each clinical feature was similar regardless of whether individuals were homozygous for the *SMN1* deletion or were compound heterozygous (*SMN1* deletion and one P/LP *SMN1* variant or one VUS) (Figure 2B). In addition, an assessment of eight pedigrees that included individuals who were compound heterozygotes demonstrated that their disease onset was similar to that in individuals with homozygous deletions (Figure 3), of which six families had phase confirmed (Figure 3, Families A, B, C, E, F, and H). For the two families in which phasing could not be confirmed, there was an affected sibling with the same genotype as the proband (families D and G) suggesting the variants segregated with disease.

In addition, nine individuals with two copies of *SMN1* were found to have sequence variants (Supplementary Table 2), eight of whom were categorized as indeterminate VUS and one who was categorized as a carrier.

### Influence of *SMN2* Copy Number on Age at Diagnosis and Symptom Severity

**Figure 4:**
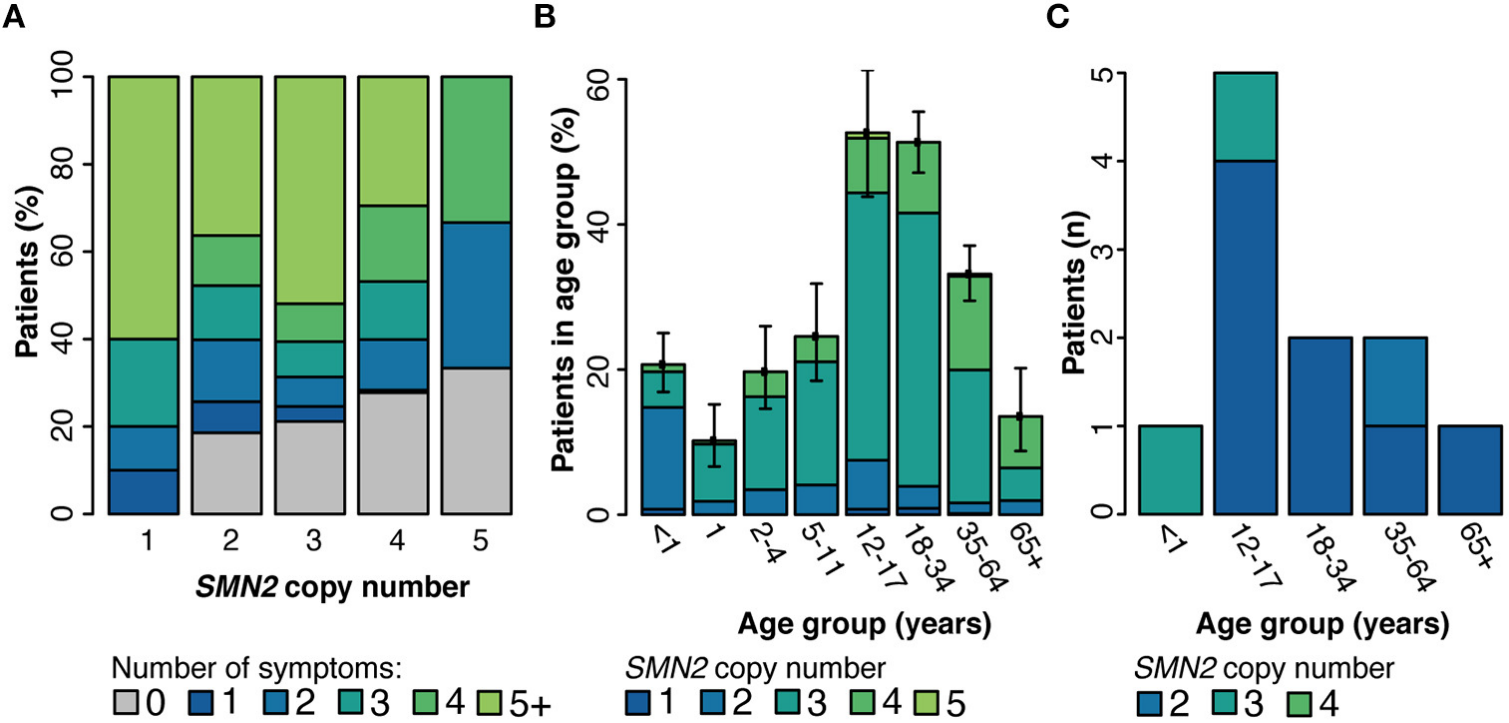
Genetic modifiers of SMA. Among individuals with a positive molecular diagnosis who underwent testing through the SMA Identified Program, the number of clinician-reported symptoms **(A)** and age distribution **(B)** were reported by *SMN2* copy number. **(C)** Among all individuals with a positive diagnosis who were tested through Invitae (SMA Identified or outside of the program), those with the *SMN2* c.859G>C variant were stratified by age at time of testing and *SMN2* copy number. 95% confidence intervals for every subgroup in panels **(A,B)** are reported in Supplementary Tables 3, 4 respectively.

The association of *SMN2* copy number with both age at molecular diagnosis and clinician-reported symptoms was explored. A range of one to six copies of *SMN2* were observed in individuals with a positive molecular diagnosis tested through SMA Identified. The number of symptoms was inversely correlated with *SMN2* copy number. Nearly 60% of individuals with diagnostic/nearly diagnostic results with only one copy of *SMN2* reported at least five symptoms, whereas, one-quarter of those with four or more copies of *SMN2* reported no symptoms (Figure 4A). When stratified according to age, most individuals (70%) with a positive diagnosis were under 34 years of age. *SMN2* copy number was positively correlated with age at the time of genetic testing (Spearman's rho = 0.41, *p* = 1.89E-32) (Figure 4B).

### Correlation Between the *SMN2* c.859G>C Modifier Allele and Age at Testing and *SMN2* Copy Number

Although there are no clinical guidelines for reporting the *SMN2* c.859G>C variant in the course of genetic testing, we assessed its effect on the age of disease onset and in relation to *SMN2* copy number. Among probands tested within or outside of SMA Identified, the *SMN2* c.859G>C variant was observed in 0.8% of individuals (*n* = 11/1,345) with a positive SMA molecular diagnosis. Among probands with a positive SMA molecular diagnosis, those with the modifier were on average older at testing with lower *SMN2* copy number, although the modifier was not found to significantly impact age at testing when controlling for copy number (two-way ANOVA, Figure 4C). When clinical information was reported for these individuals (*n* = 8), clinician-reported symptoms ranged from asymptomatic to muscle weakness, tendon reflexes, scoliosis, joint contractures, and tongue fasciculations.

## Discussion

The goals of this study were to assess the efficacy of a sponsored testing program in diagnosing SMA, to summarize the clinical features among individuals who underwent testing for SMA, and to characterize the relationships between the molecular etiologies and the clinical presentation of SMA. Nearly one-third (31.3%) of unrelated individuals tested through SMA Identified received a positive molecular diagnosis, demonstrating that a sponsored testing program is an effective approach for providing molecular diagnoses among individuals with a suspected or confirmed clinical diagnosis of SMA. In characterizing the clinical features among individuals, we found that although muscle weakness was most commonly reported, it was not as predictive of a positive molecular diagnosis as was the presence of spinal rods, spinal fusions, and scoliosis. These findings may provide clinicians additional guidance to systematically ascertain presenting symptoms that warrant genetic testing for SMA, which has important implications in the era of available therapies for SMA (26). Additionally, we observed that *SMN2* copy number and the *SMN2* c.859G>C allele modify clinical severity, as measured by the number of reported symptoms and age at the time of testing.

Similar to other reports (4), our study found that the majority (96.2%) of positive diagnoses was attributed to a homozygous deletion of *SMN1*, with the remaining positive diagnoses accounted for by compound heterozygous sequence variants. The clinical features associated with compound heterozygous individuals have been reported in a handful of case reports and small cohort studies, demonstrating that disease severity may vary based on the sequence variant (27–31). In this study, we identified 29 (3.8%) of 771 individuals with one *SMN1* deletion and one sequence variant. Of these, two individuals had a diagnostic result with the sequence variant confirmed to be in *SMN1*, and 27 had variants with locations that could not be disambiguated. The reported clinical symptoms for these 29 compound heterozygous individuals were similar to those for individuals homozygous for the *SMN1* deletion. Although the disease presentation may be similar between these two groups, compound heterozygous children would be missed by newborn screening programs, which use genotyping technologies that cannot identify sequence variants (32, 33).

Among individuals with a positive SMA diagnosis, *SMN2* copy number and the rare *SMN2* c.859G>C allele can explain disease severity and inform therapeutic options. Here, we identified 11 patients with a diagnostic result who also had the *SMN2* c.859G>C variant. Consistent with previous studies, we found that age at testing was older in these patients than in others and was independent of *SMN2* copy number (7, 8). Although our sample size was limited because of the rarity of the *SMN2* c.859G>C variant, the findings here provide additional evidence that this variant reduces clinical severity. Although the detection of this modifier has been used primarily in the context of excluding individuals with mild phenotypes from SMA clinical trials assessing the safety and efficacy of disease-modifying agents, the broader utility of detecting this modifier is unknown. Further genotype-phenotype studies may provide additional evidence of the clinical impact of c.859G>C on SMA disease severity.

Clinical trials and novel therapies for rare diseases often require a positive molecular diagnosis for eligibility to participate. A key eligibility criterion for access to FDA-approved SMA treatments as well as qualification for clinical trials is confirmation *via* genetic testing of a homozygous deletion or compound heterozygous mutation of *SMN1*. Thus, all individuals in this study with diagnostic results would be eligible to seek treatment with an FDA approved drug for SMA or to participate in a clinical trial for this disease. For nearly diagnostic results, patients could still be eligible for FDA approved drugs or clinical trials if their sequence variant is disambiguated externally by LR-PCR and confirmed as pathogenic. Unfortunately, the scope of this project did not allow confirmation of eligibility for approved treatments or inclusion into clinical trials. However, this study highlights that sequence variants do contribute to the pathogenesis of SMA and that detection of a VUS using our methods should not be a barrier to seeking time-sensitive treatments for SMA, but rather they should prompt additional efforts to disambiguate the variants and to help confirm or rule out a diagnosis of SMA. Precisely because access to therapies hinges on molecular diagnoses, it is essential to use language in clinical reporting that specifies the pathogenicity of variants if they were to be disambiguated, even if their disambiguation is not possible by short-read NGS. Inclusion of this information in diagnostic reports can support the need for, and justification of, any additional confirmatory testing to patients, their clinicians, and their insurers.

In 2010, the National Institutes of Health initiated the Therapeutics for Rare and Neglected Diseases program to aid in bringing together multidisciplinary stakeholders to improve the drug development process for such rare diseases (34). This initiative has generated several collaborations designed to address this challenge. In addition, collaborative partnerships among clinicians, genetic diagnostic laboratories, and biopharmaceutical companies offering access to diagnostic genetic testing represent a powerful new paradigm in clinical genomics that can facilitate access to clinical care for individuals afflicted by rare diseases such as SMA. Indeed, our study of SMA Identified demonstrates the utility of such partnerships through a high diagnostic yield and effective identification of SMA patients. This may be attributed to increased awareness of the program as well as the fact that it removes financial barriers to accessing genetic testing.

Although not unique to this study, the results reported here must be interpreted in the context of the limitations of analyzing *SMN1* and *SMN2* by NGS. The ability to determine whether a sequence variant detected by NGS occurs in *SMN1* or *SMN2* relies on its proximity to the gene-determining variant in exon 7. Although a nearly diagnostic result supported a diagnosis of SMA in 27 individuals, these findings would require additional disambiguation by LR-PCR (35), which is not employed by our laboratory for SMA and must be pursued externally. Despite these limitations, phasing by family variant testing for nearly diagnostic sequence variants strongly supports that they are causal (Figure 3, Supplementary Table 1). In addition, variants exhibiting mosaicism or chromosomal rearrangements may not be detected, and determining the zygosity of ambiguous sequence variants based on variant allele frequency alone may not be possible (36). Furthermore, primer or probe binding regions that overlap with variants may influence analytical and clinical specificity by interfering with variant confirmation (37). RNA studies could be used in situations where NGS and confirmatory testing yield contradictory results. In addition, caution should be taken in interpreting the clinician-provided clinical features most commonly observed among the various genetic test results. Clinicians may have only provided the minimal required information to make their patients eligible for testing through the sponsored program. To account for this, the diagnostic yield was calculated by clinical feature only for those with that feature noted. However, we do note that nearly two-thirds of individuals in the cohort had at least two clinical features noted, suggesting that clinicians did provide comprehensive details on their patients' clinical presentation. Further studies with more complete clinical information will help to clarify the findings presented here.

Identifying SMA early through clinical features and confirming the diagnosis through genetic testing may have considerable impact by accelerating access to clinical care and reducing SMA-associated morbidity in affected individuals. Ongoing analysis of individuals tested through the SMA Identified program may provide additional insights into the clinical presentations most associated with a positive diagnosis, the genetic landscape of sequence variants in compound heterozygotes, and the influence of genetic modifiers on disease severity. Follow-up studies investigating the utility of sponsored programs in providing earlier access to therapy for SMA are required.

## Data Availability Statement

The original contributions presented in the study are included in the article/Supplementary Material, further inquiries can be directed to the corresponding authors.

## Ethics Statement

The studies involving human participants were reviewed and approved by Western Institutional Review Board. Written informed consent from the participants' legal guardian/next of kin was not required to participate in this study in accordance with the national legislation and the institutional requirements.

## Author Contributions

BMB interpreted variants, designed analyses, prepared figures, and wrote the paper. RT managed the patient database, retrieved data for statistical analyses, performed analyses, and prepared figures. SLB prepared tables and wrote the paper. AM provided critical review of the paper. CAT, MJW, SA, BAJ, TLW, and JC conceived of the paper, edited the paper, and reviewed variant interpretations. JC led the Biogen-funded study. All authors contributed to the article and approved the submitted version.

## Conflict of Interest

BMB, RT, SA, SLB, BAJ, AM, CAT, MJW, and TLW are employees and shareholders of Invitae, and JC is an employee and shareholder of Biogen.
