# Supplementary material for: SMA Identified: Clinical and Molecular Findings From a Sponsored Testing Program for Spinal Muscular Atrophy in More Than 2,000 Individuals
Source: Front Neurol. 2021 May 6;12:663911. doi: 10.3389/fneur.2021.663911 (PMC8134668; doi:10.3389/fneur.2021.663911)
Supplement: Supplementary file 1 [file Data_Sheet_1.pdf]

Supplement to: SMA Identified: Clinical and Molecular Findings from a Sponsored Testing Program for Spinal Muscular Atrophy in More than 2,000 Individuals

Supplemental Figure 1: Age distribution of individuals tested through SMA Identified

Supplemental Figure 2: Clinician-Reported Clinical Features, Stratified by Age

Supplemental Table 1: Distribution of Sequence Variants Detected in Individuals with Diagnostic and Nearly Diagnostic Results (SMN1 copy number = 1)

Supplemental Table 2: Distribution of sequence variants detected in individuals with indeterminate results (single ambiguous sequence variant in SMN1 or SMN2, and SMN1 copy number = 2)

Supplemental Table 3: Confidence intervals for SMN2 copy number stratified by number of clinician-reported symptoms

Supplemental Table 4: Confidence intervals for *SMN2* copy number stratified by age at testing

Supplemental Figure 1. Age distribution of individuals tested through SMA Identified.

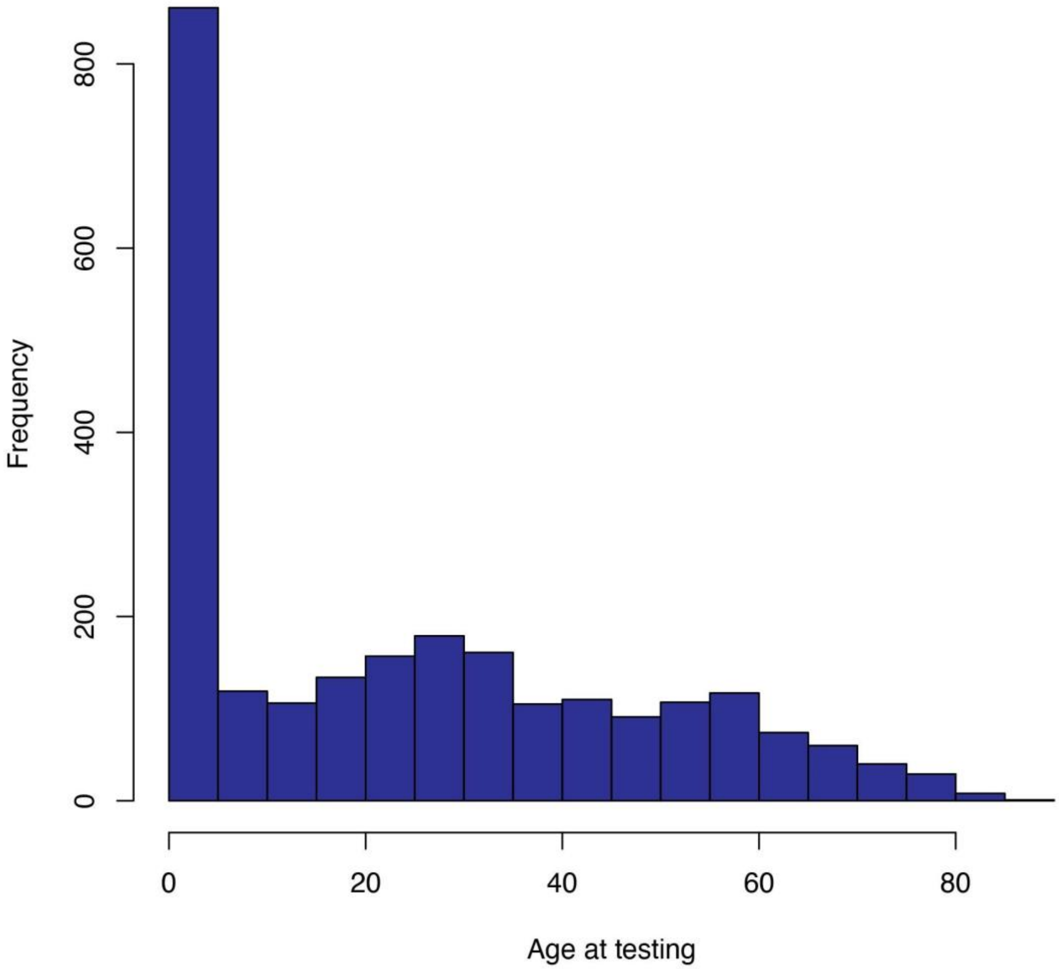

Supplemental Figure 2. Clinician-Reported Clinical Features, Stratified by Age

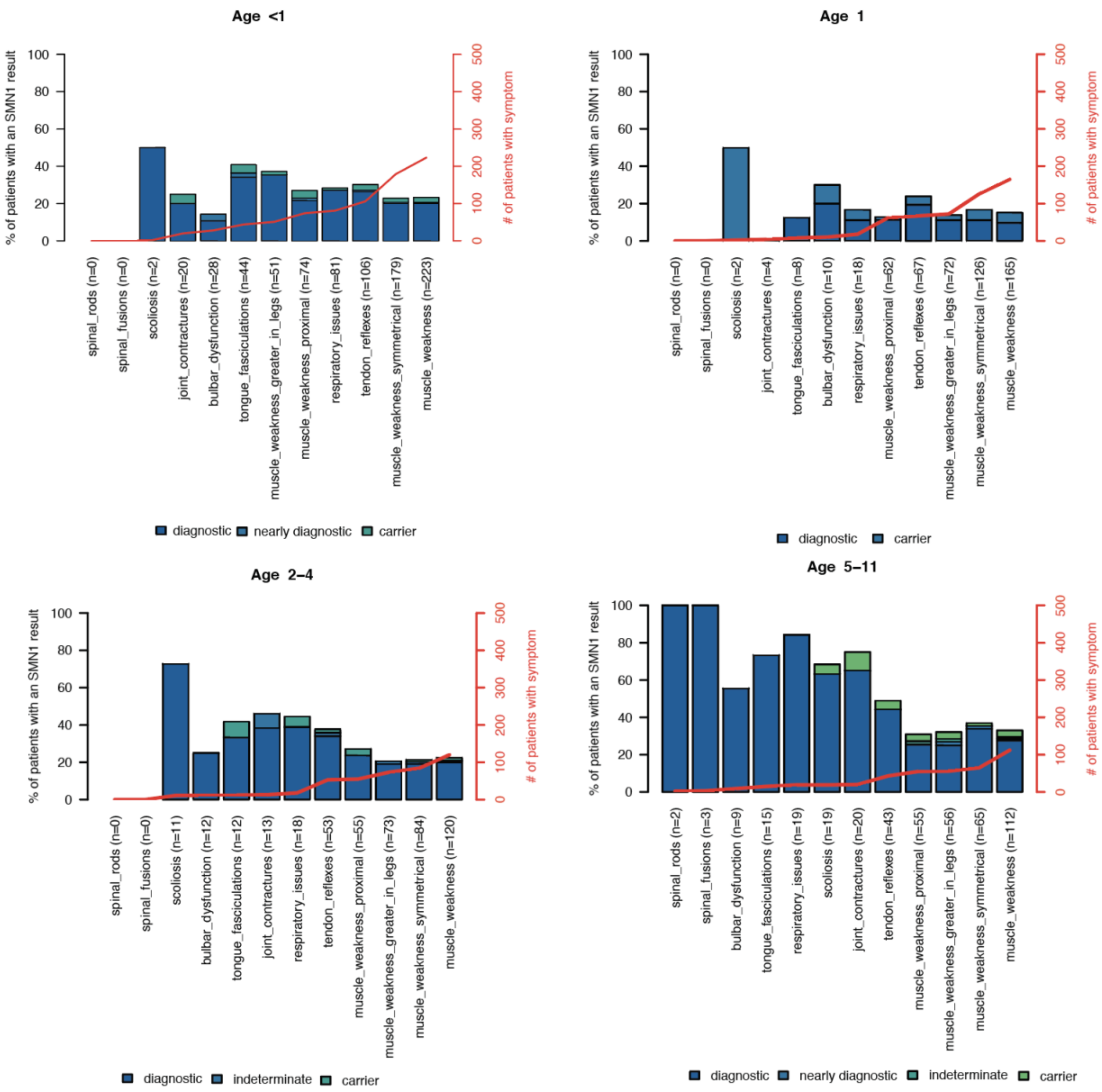

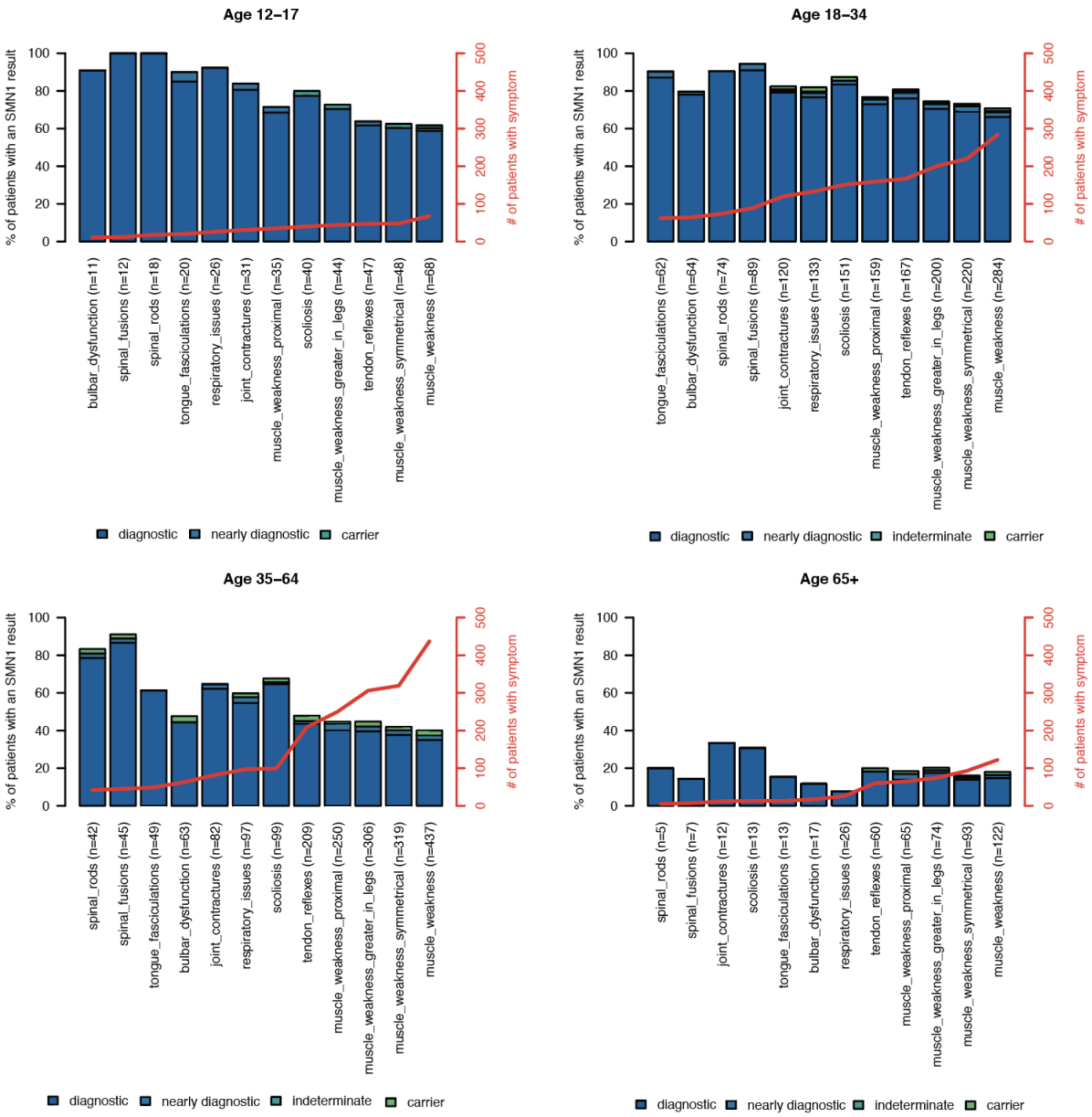
Age 12–17
% of patients with an SMN1 result
# of patients with symptom
bulbar_dysfunction (n=11)
spinal_fusions (n=12)
spinal_rods (n=18)
tongue_fasciculations (n=20)
respiratory_issues (n=26)
joint_contractures (n=31)
muscle_weakness_proximal (n=35)
scoliosis (n=40)
muscle_weakness_greater_in_legs (n=44)
tendon_reflexes (n=47)
muscle_weakness_symmetrical (n=48)
muscle_weakness (n=68)
diagnostic
nearly diagnostic
carrier
Age 18–34
% of patients with an SMN1 result
# of patients with symptom
tongue_fasciculations (n=62)
bulbar_dysfunction (n=64)
spinal_rods (n=74)
spinal_fusions (n=89)
joint_contractures (n=120)
respiratory_issues (n=133)
scoliosis (n=151)
muscle_weakness_proximal (n=159)
tendon_reflexes (n=167)
muscle_weakness_greater_in_legs (n=200)
muscle_weakness_symmetrical (n=220)
muscle_weakness (n=284)
diagnostic
nearly diagnostic
indeterminate
carrier
Age 35–64
% of patients with an SMN1 result
# of patients with symptom
spinal_rods (n=42)
spinal_fusions (n=45)
tongue_fasciculations (n=49)
bulbar_dysfunction (n=63)
joint_contractures (n=82)
respiratory_issues (n=97)
scoliosis (n=99)
tendon_reflexes (n=209)
muscle_weakness_proximal (n=250)
muscle_weakness_greater_in_legs (n=306)
muscle_weakness_symmetrical (n=319)
muscle_weakness (n=437)
diagnostic
nearly diagnostic
indeterminate
carrier
Age 65+
% of patients with an SMN1 result
# of patients with symptom
spinal_rods (n=5)
spinal_fusions (n=7)
joint_contractures (n=12)
scoliosis (n=13)
tongue_fasciculations (n=13)
bulbar_dysfunction (n=17)
respiratory_issues (n=26)
tendon_reflexes (n=60)
muscle_weakness_proximal (n=65)
muscle_weakness_greater_in_legs (n=74)
muscle_weakness_symmetrical (n=93)
muscle_weakness (n=122)
diagnostic
nearly diagnostic
indeterminate
carrier

Supplemental Table 1. Distribution of Sequence Variants Detected in Individuals with Diagnostic and Nearly Diagnostic Results (SMN1 copy number = 1)

					Phase		
**Variant[a]**	**Effect**	**Interpretation**	**ClinVar ID**	**Probands with variant**	**Likely trans by family testing**	**In trans by NGS**	**Not resolved**
SMN1 c.835-3C>T	Intronic	Pathogenic	495829	1		1[b]	NA
SMN1 c.835-18_835-12del	Intronic	Likely Pathogenic	pending submission	1		1[b]	NA
SMN1 or *SMN2* c.5C>G	p.Ala2Gly	VUS[c]	9168	6	1		5
SMN1 or *SMN2* c.305G>A	p.Trp102*	VUS[c]	9173	1	1		NA
SMN1 or *SMN2* c.788T>C	p.Met263Thr	VUS	pending submission	2			2
***SMN1* or *SMN2* c.275G>A**	**p.Trp92***	**VUS[c]**	pending submission	1			1
***SMN1* or *SMN2* c.475-2A>T**	**Splice acceptor**	**VUS[c]**	pending submission	2			2
SMN1 or *SMN2* c.821C>T	p.Thr274Ile	VUS[c]	9164	2	1		1
***SMN1* or *SMN2* c.628-1delG**	**Splice acceptor**	**VUS[c]**	pending submission	1			1
SMN1 or	p.Ser230L	VUS[c]	pending	1			1

SMN2 c.689C>T	eu		submission				
SMN1 or *SMN2* c.785G>T	p.Ser262Ile	VUS[c]	9165	1			1
SMN1 or *SMN2* c.770_780 dupCTGA TGCTTTG	p.Gly261Le ufs*8	VUS[c]	586627	2	1		1
SMN1 or *SMN2* c.824G>T	p.Gly275V al	VUS	pending submission	2	1		1
***SMN1* or *SMN2* c.419A>T**	**p.Asp140V al**	**VUS**	**448428**	**2**	1	1[d]	NA
SMN1 or *SMN2* c.815A>G	p.Tyr272C ys	VUS[c]	9166	1	1		NA
SMN1 or *SMN2* c.93_96du p	p.Ile33*	VUS[c]	448443	2	1		1
SMN1 or *SMN2* c.510_511 del	p.Ser170Ar gfs*9	VUS[c]	634941	1			1

[a]Bolded rows indicate novel variants. [b]Unambiguously in SMN1 by IGV, NGS reads were in proximity of the informative gene-determining variant. [c]Pathogenic or Likely Pathogenic when unambiguously in SMN1, VUS when disambiguation not possible by NGS. [d]Unambiguously confirmed by LR-PCR externally pursued by the individual.

Supplemental Table 2. Distribution of sequence variants detected in individuals with indeterminate results (single ambiguous sequence variant in SMN1 or SMN2, and SMN1 copy number greater than or equal to 2)

				Molecular diagnosis
**Variant[a]**	**Effect**	**Interpretation**	**ClinVar Variation ID**	**Indeterminate VUS (Number of Probands)**
***SMN1* c.835-2A>C**	**Splice acceptor**	**Likely Pathogenic[b]**	pending submission	**1**
SMN1 or *SMN2* c.5C>G	p.Ala2Gly	VUS[c]	9168	2
***SMN1* or *SMN2* c.275G>A**	**p.Trp92***	**VUS[c]**	pending submission	**1**
SMN1 or *SMN2* c.770_780dupCTGATGCTTTG	p.Gly261Leufs*8[d]	VUS[c]	586627	1
SMN1 or *SMN2* c.824G>T	p.Gly275Val[d]	VUS	pending submission	1
***SMN1* or *SMN2* c.321C>A**	**p.Cys107***	**VUS[c]**	pending submission	**1**
***SMN1* or *SMN2* c.346A>G**	**p.Ile116Val**	**VUS**	pending submission	**1**
***SMN1* or *SMN2* c.689C>A**	**p.Ser230*[e]**	**VUS[c]**	pending submission	**1**

[a]Bolded rows indicate novel variants.

[b]Unambiguously in SMN1 by IGV, but no second variant identified

[c]Pathogenic or Likely Pathogenic when unambiguously in SMN1, VUS when disambiguation not possible by NGS

[d]This variant was likely trans from an SMN1 deletion in an unrelated proband in Table 1

[e]Variant allele frequency strongly suggests this individual is homozygous for this variant. Notably, parents were consanguineous, but did not pursue family variant testing.

Supplemental Table 3. Confidence intervals for *SMN2* copy number stratified by number of clinician-reported symptoms[a]

	***SMN2* copy number**				
% (95% CI)	**1 copy**	**2 copies**	**3 copies**	**4 copies**	**5 copies**
0 symptoms	0 (0-34.45)	18.8 (12.12-27.23)	21.2 (17.64-25.21)	27.7 (21.35-35.15)	33.3 (1.77-87.47)
1 symptom	10.0 (0.52-45.88)	7.1 (3.33-13.90)	3.4 (2.02-5.56)	0.6 (0.030-3.67)	0 (0-69.00)
2 symptoms	10.0 (0.52-45.88)	14.2 (8.56-22.27)	6.8 (4.75-9.54)	11.6 (7.38-17.51)	33.3 (1.77-87.47)
3 symptoms	20.0 (3.54-55.78)	12.4 (7.19-20.24)	8.1 (5.83-10.98)	13.3 (8.79-19.49)	0 (0-69.00)
4 symptoms	0 (0-34.45)	11.5 (6.51-19.21)	8.7 (6.38-11.69)	17.3 (12.18-23.99)	33.3 (1.77-87.47)
5+ symptoms	60.0 (27.37-86.31)	36.3 (27.60-45.91)	51.9 (47.30-56.49)	29.5 (22.93-36.96)	0 (0-69.00)

Abbreviations: CI, confidence interval.

[a]Corresponding to Figure 4A in the main article.

Supplemental Table 4. Confidence intervals for *SMN2* copy number stratified by age at testing[a]

	***SMN2* copy number**				
% (95% CI)	**1 copy**	**2 copies**	**3 copies**	**4 copies**	**5 copies**
<1 year	0.7 (0.19-2.33)	14.0 (10.89-17.89)	4.9 (3.11-7.63)	1.0 (0.32-2.68)	0 (0-1.17)
1 year	0 (0-2.18)	1.9 (0.59-4.99)	7.9 (4.79-12.51)	0.5 (0.024-2.95)	0 (0-2.18)
2-4 years	0 (0-2.31)	3.4 (1.52-7.27)	12.8 (8.69-18.38)	3.4 (1.52-7.27)	0 (0-2.31)
5-11 years	0 (0-2.74)	4.1 (1.81-8.58)	17.0 (11.83-23.62)	3.5 (1.43-7.83)	0 (0-2.74)
12-17 years	0.8 (0.039-4.74)	6.8 (3.34-12.83)	36.8 (28.77-45.68)	7.5 (3.87-13.75)	0.8 (0.039-4.74)
18-34 years	0.9 (0.33-2.18)	3.0 (1.83-4.89)	37.7 (33.66-41.82)	9.8 (7.50-12.60)	0 (0-0.84)
35-64 years	0.2 (0.0085-1.05)	1.5 (0.72-2.88)	18.3 (15.36-21.65)	12.9 (10.41-15.89)	0.3 (0.057-1.31)
65+ years	0 (0-3.01)	1.9 (0.50-6.00)	4.5 (1.99-9.44)	7.1 (3.77-12.65)	0 (0-3.01)

Abbreviations: CI, confidence interval.

[a]Corresponding to Figure 4B in the main article.
